# Human Metapneumovirus and Chronic Obstructive Pulmonary Disease

**DOI:** 10.3201/eid1007.030633

**Published:** 2004-07

**Authors:** Diego Vicente, Milagrosa Montes, Gustavo Cilla, Emilio Pérez-Trallero

**Affiliations:** * Hospital Donostia, San Sebastián, Spain;; † Basque Country University, San Sebastián, Spain

**Keywords:** letter, Metapneumovirus, SARS, SARS-CoV, respiratory infection

**To the Editor:** We read with interest an article, Human Metapneumovirus Detection in Patients with Severe Acute Respiratory Syndrome, in your journal (1). In the report, Chan et al., did not question that SARS-CoV is the etiologic agent of severe acute respiratory syndrome (SARS); however, human metapneumovirus (HMPV) was found in 25 (52%) of 48 probable SARS cases that were investigated, and SARS-CoV was detected in 11 (22.9%) of them. Another recent article reported HMPV in five of six patients in whom SARS was diagnosed in Canada (2); four of the six were coinfected with SARS-CoV. The prevalence of HMPV infection in SARS patients validates the interest in HMPV’s possible role in SARS etiology.

From November 2001 to February 2002, 1 year before the first cases of SARS appeared, we tested the sputum of patients >64 years of age who had experienced exacerbation of chronic obstructive pulmonary disease, for HMPV. Investigations were conducted on 90 episodes in 89 elderly patients, 62 males and 27 females, in which we found no other microorganisms that could have been related to the exacerbation of chronic obstructive pulmonary disease. RNA was extracted from the sputum samples and amplified by reverse transcriptase–polymerase chain reaction (RT-PCR) to detect HMPV as previously described (3). Results of bacterial culture and culture and PCR to detect respiratory syncytial virus and influenza virus types A and B were negative, whereas HMPV was found in the sputum of five (three men and two women) immunocompetent patients, 77–87 years of age. The prevalence of HMPV infection was 5.5%, similar to the percentage obtained by Chan et al., when HMPV RT-PCR was conducted on the respiratory samples. Fever (temperature >38°C) was not present in any of the five patients infected with HMPV. Two patients were admitted to a hospital. Both patients had bronchial infection and cough with bronchospasm and moderate respiratory insufficiency (oxygen saturation rate: 90.3% and 88%, respectively) for >1 week. Sputum samples from an additional 70 elderly patients with exacerbation of chronic obstructive pulmonary disease with positive detection for influenza virus (n = 50) or respiratory syncytial virus (n = 20) were tested for HMPV infection. None of the samples showed HMPV infection.

Sequence analysis of amplicons from the five samples positive for HMPV infection showed >95% similarity with HMPV sequences found in other parts of the world (4,5). Additional studies should be conducted to confirm that HMPV exacerbates chronic obstructive pulmonary disease. However, by performing an RT-PCR directly on the sample instead of the more efficient RT-PCR after viral culture used by Chan et al., these findings suggest that HMPV is a frequently undetected agent in acute respiratory infection unrelated to SARS. The important questions are whether HMPV and SARS-CoV coinfection would facilitate more severe SARS, or whether HMPV infection would facilitate a more efficient transmission of SARS-CoV.
